# Pharmacokinetics of Ketoprofen in Nile Tilapia (*Oreochromis niloticus*) and Rainbow Trout (*Oncorhynchus mykiss*)

**DOI:** 10.3389/fvets.2020.585324

**Published:** 2020-10-07

**Authors:** Whitney Greene, Natalie D. Mylniczenko, Timothy Storms, Charlene M. Burns, Gregory A. Lewbart, Lynne Byrd, Mark G. Papich

**Affiliations:** ^1^Mote Marine Laboratory and Aquarium, Sarasota, FL, United States; ^2^John G. Shedd Aquarium, Chicago, IL, United States; ^3^Woodland Park Zoo, Seattle, WA, United States; ^4^College of Veterinary Medicine, North Carolina State University, Raleigh, NC, United States

**Keywords:** ketoprofen, teleost, tilapia, rainbow trout, pharmacokinctics, non-steroidal anti-inflammatory drugs (NSAIDs), *Oncorhynchus mykiss*, *Oreochromis niloticus*

## Abstract

The objective of this study was to document the pharmacokinetics of ketoprofen following 3 mg/kg intramuscular (IM) and intravenous (IV) injections in rainbow trout (*Oncorhynchus mykiss*) and 8 mg/kg intramuscular (IM) injection in Nile tilapia (*Oreochromis niloticus)*. Plasma was collected laterally from the tail vein for drug analysis at various time intervals up to 72 h following the injection of ketoprofen. In trout, area under the curve (AUC) levels were 115.24 μg hr/mL for IM and 135.69 μg hr/mL for IV groups with a half-life of 4.40 and 3.91 h, respectively. In both trout and tilapia, there were detectable ketoprofen concentrations in most fish for 24 h post-injection. In tilapia, there was a large difference between the R- and S-enantiomers, suggesting either chiral inversion from R- to S-enantiomer or more rapid clearance of the R-enantiomer. AUC values of the S- and R-enantiomers were 510 and 194 μg hr/Ml, respectively, corresponding to a faster clearance for the R-enantiomer. This study shows that there were very high plasma concentrations of ketoprofen in trout and tilapia with no adverse effects observed. Future studies on the efficacy, frequency of dosing, analgesia, adverse effects, and route of administration are warranted.

## Introduction

There is increasing awareness to the importance of animal welfare in teleost fish under human care, with particular attention to pain and inflammation management. Previously, references suggested that fish are incapable of feeling pain (1), but further research has elucidated that fish possess nociceptors which transmit painful stimuli and are similar to nociceptors found in mammals (2–6). Additionally, teleosts have been found to respond adversely to negative stimuli and such corresponding behavioral changes may indicate a response to pain (7–11). With this increased knowledge in teleost pain response, fish may benefit from medications that aid in reducing pain and inflammation (12–16). Two studies in the Netherlands support this claim and concluded that humane medical practice dictates the incorporation of appropriate analgesics into medical treatments and clinical procedures for fish (17, 18).

Ketoprofen functions by inhibiting arachidonate cyclooxygenase (COX) enzymes, thereby reducing the production of thromboxanes and prostaglandins. Teleosts possess COX enzymes identical to those of mammals (18–20) and in mammals, the inhibition of these enzymes results in anti-inflammatory, antipyretic, and analgesic properties. Therefore, fish should benefit from anti-inflammatory and analgesic drugs (21). Ketoprofen is a racemic mixture of R- and S- enantiomers (22, 23). Although the two enantiomers have the same chemical structure, they produce differences in pharmacology, toxicology, pharmacokinetics, and metabolism (24). In mammals, the S-form is the most active for therapeutic effects (eutomer); however, it is unknown which enantiomer is the most active in ectotherms (24). In order to provide the needed analgesia and anti-inflammation, more information about how these medications influence teleosts is needed to establish dosages and treatment regimens.

There have been three studies examining analgesic effect of ketoprofen in fish but no pharmacokinetic data has been reported (12, 14, 25). Pharmacokinetics have been reported for meloxicam in tilapia, but the half-life (IV = 1.36 h and IM = 1.8 h), and elimination time were both rapid compared to dogs (24 h) (26), therefore a different drug in this species is warranted (27). Pharmacokinetic data for ketoprofen is essential to understand its disposition and to facilitate the design of dosing regimens. To this end, the objective of this study was to generate pharmacokinetic data for ketoprofen use in rainbow trout (*Oncorhynchus mykiss*) and Nile tilapia (*Oreochromis niloticus)*. The intravenous route is recommended to generate pharmacokinetics in exotic species (28), however, intramuscular is often more practical clinically and less stressful to the animal. Ketoprofen was administered by injection, via intravenous (IV) and intramuscular (IM) in trout at 3 mg/kg body weight and via IM injection at 8 mg/kg in tilapia to establish pharmacokinetics.

## Materials and Methods

### Animal Husbandry (Trout)

Twenty-four aquaculture-raised trout were included in the study, with an average weight of 819 g for the animals in the IV injection group and 775 g for the IM injection group. Animals were kept in individually labeled floating laundry baskets measuring 68 × 47 × 30 cm inside a larger tank for the study. Animals were allowed to acclimate for a minimum of 30 days prior to the initiation of the study. All individuals were determined to be clinically healthy based on behavior and body condition, with no visible signs of illness or disease. Water temperature was maintained at 15°C and water quality was kept within ranges considered appropriate for the species. The study was approved by the research committee at the John G. Shedd Aquarium, Chicago, Illinois.

### Animal Husbandry (Tilapia)

Twelve Nile tilapia were collected from a larger population of fish that had no visible signs of illness and were determined to be clinically healthy based on behavior and body condition. The average weight for the fish was 733 g. Animals were kept in individually labeled floating laundry baskets measuring 68 × 47 × 30 cm inside a larger 6,814 L fiberglass closed recirculating tank for the study. Animals were acclimated for 36 h prior to the initiation of the study. Water was maintained at 28°C and all water quality parameters were kept within ranges appropriate for the species. The study was approved by Mote Marine Laboratory's Institutional Animal Care and Use Committee (IACUC).

### Sample Collection (Trout)

Twenty-four trout were divided into two groups of 12 animals each; at each session, animals were gently moved into a net from the basket and anesthetized with tricaine methanesulfonate (MS-222; Argent Chemical Laboratories, Redmond, WA 98052, USA) at 100 mg/L concentration, procedures usually lasted 3–5 min. Each group of 12 animals received an IV or IM injection of ketoprofen (Ketofen 100 mg/ml injectable solution, Zoetis Inc., Kalamazoo, MI 49007, USA) at 3 mg/kg. This dosage was determined from an initial study looking at analgesia and minimum gill concentration (MGC) with MS-222 and ketoprofen with dosages tested from 1 to 5 mg/kg in 1 mg/kg increments (14).

Blood samples (0.5–1.0 mL) were collected from the caudal tail vein using 22-ga needles attached to 3-mL syringes and placed in lithium heparin (Li-heparin) microtainers. Six animals in each injection group had samples of blood collected at: 0, 0.5, 3, 12, 48, and 96 h, while the other six fish were sampled at: 0,1.5, 6, 24, and 72 h. All blood samples were centrifuged within 30 min after each collection at 700 relative centrifugal field (RCF) for 15 min. Plasma was then collected via pipette from each sample and stored in cryovials at −80°C until analysis. Animals were returned to the animal collection after the study, with no negative side effects identified.

### Sample Collection (Tilapia)

All 12 tilapia were administered a single IM injection of ketoprofen (Ketofen 100 mg/ml injectable solution, Zoetis Inc., Kalamazoo, MI 49007, USA) at 8 mg/kg without anesthesia. Animals were moved into a net from the basket, wrapped in a chamois cloth, and venipuncture performed all within 60 s. On rare occasions a second attempt required, this extended the handling time an additional 15–30 s. The above dosage was based on results from a pilot study in spadefish (*Chaetodipterus faber*) at dosages of 1, 2, 4, and 8 mg/kg IM, and the above mentioned trout pilot study (14). Blood samples (0.5–1.0 mL) were collected from the caudal tail vein using 22-ga needles attached to 3-mL syringes by a trained aquatic animal veterinary technician to minimize handling time and stress at varying time points (0, 0.5, 1, 2, 4, 8, 12, 24 h) and placed in 1.3 ml lithium heparin (Li-heparin) microtainers. Some minor skin darkening was observed around the injection site in 2 of the 12 animals sampled, but this resolved after the 1 h sampling point.

All blood samples were centrifuged within 15 min after each collection at 1372 RCF for 5 min. Plasma was then collected via pipette from each sample and banked in cryovials at −80°C until analysis. Following the final sample collection, all fish were humanely euthanized using MS-222 (Syndel, Ferndale, WA 98248, USA) at 750 ppm for 30 min followed by severing of the spinal cord.

### Sample Processing and Analysis (Trout)

Plasma samples were analyzed using a reverse phase high-performance liquid chromatography method. The system consisted of a 2,690 separations module, a 2,487 absorbance detector, and a computer equipped with Empower software. Ketoprofen was extracted from plasma samples using liquid extraction. Previously frozen samples were briefly thawed and vortexed. One milliliter of plasma was transferred to a clean screw-top test tube followed by 75 μL internal standard (5.0 μg/mL phenacetin). One hundred microliters of 3% formic acid was added followed by 6 mL of chloroform: IPA (80:20) and the tubes were rocked for 15 min and then centrifuged for 15 min at 1,000 × g. The resulting layer was transferred to a clean tube and evaporated to dryness with nitrogen gas.

The compounds were separated on a Symmetry Shield RP 18 (3.9 × 150 mm, 5 μm) column with a Symmetry Shield RP 18 guard column (Waters Corporation, Milford, MA 01757, USA). The mobile phase was an isocratic mixture of (A) 20 mM potassium dihydrogen phosphate adjusted to pH 3.0 and (B) methanol (50:50). The flow rate was 1 mL/min and the column temperature was ambient. Absorbance was measured at 258 nm.

Standard curves for plasma analysis were prepared by fortifying untreated, pooled plasma with ketoprofen to produce a linear concentration range of 10–15,00 ng/mL. Calibration samples were prepared exactly as plasma samples. Average recovery for ketoprofen was 95% while intra- and inter-assay variability were below 10%.The lower limit of quantification was 10 ng/mL.

### Sample Processing and Analysis (Tilapia)

In this study, a selective chiral assay was used to distinguish between the R- and S-isomer of ketoprofen in tilapia; when the study with rainbow trout was conducted, a chiral assay was not available. A validated assay from a previous study was performed (29) for the drug analysis of the R- and S- enantiomers of ketoprofen. Drug concentrations were determined using a high-pressure liquid chromatography (HPLC) chiral assay that separates the R- and S-enantiomers of ketoprofen. The assay for this study was validated by analyzing blank plasma from untreated fish that was fortified with concentrations of a ketoprofen reference standard. The method was accepted for this study after it was confirmed that it met acceptance criteria from the earlier study.

### Pharmacokinetic Analysis

A population pharmacokinetic approach with non-linear mixed-effects modeling was used for the trout (Phoenix®, NLME™, version 8.4, Certara, Princeton, NJ 08540, USA). This design does not allow individual pharmacokinetics parameters calculated for each animal, but it generates values for fixed effects and random effects for the population. A naïve averaged data approach was used to obtain initial estimates (data not shown). From these initial estimates, and using a single bolus input, a non-linear mixed effects (NLME) model was fitted to these data. Compartmental analysis of the data from the ketoprofen concentrations in trout was calculated using a 1-compartment model with first-order input. The model calculated the primary parameters of elimination rate constant (Ke) and volume of distribution (V). Secondary parameters calculated include the half-life, area-under-the-curve (AUC), and peak concentration (C_MAX_).

Pharmacokinetic analysis for ketoprofen administered to tilapia was performed, for each enantiomer, using a one-compartment pharmacokinetic model, with first-order input and elimination (WinNonlin™, Phoenix®, version 8.4, Certara, Princeton, NJ 08540, USA). A simple, one-compartment model provided the best fit to the data after analyzing diagnostic plots and goodness-of-fit criteria. The pharmacokinetic results were averaged for all fish using a standard two-stage (STS) approach.

Various models were tested with different error structures to determine the best-fit base model. The models were parameterized using the simplest model that provided acceptable goodness of fit plots, diagnostic plots of residuals, and scatter plots of predicted vs. observed values. Inter-individual (between-subject) variability were expressed using an exponential error model. The values calculated included a typical value (theta, or fixed effect) for the population estimate of the parameter of interest, and random effect for the inter-individual differences of the parameter of interest. An error value was included for the residual intra-subject variability.

## Results

**Figure 1:**
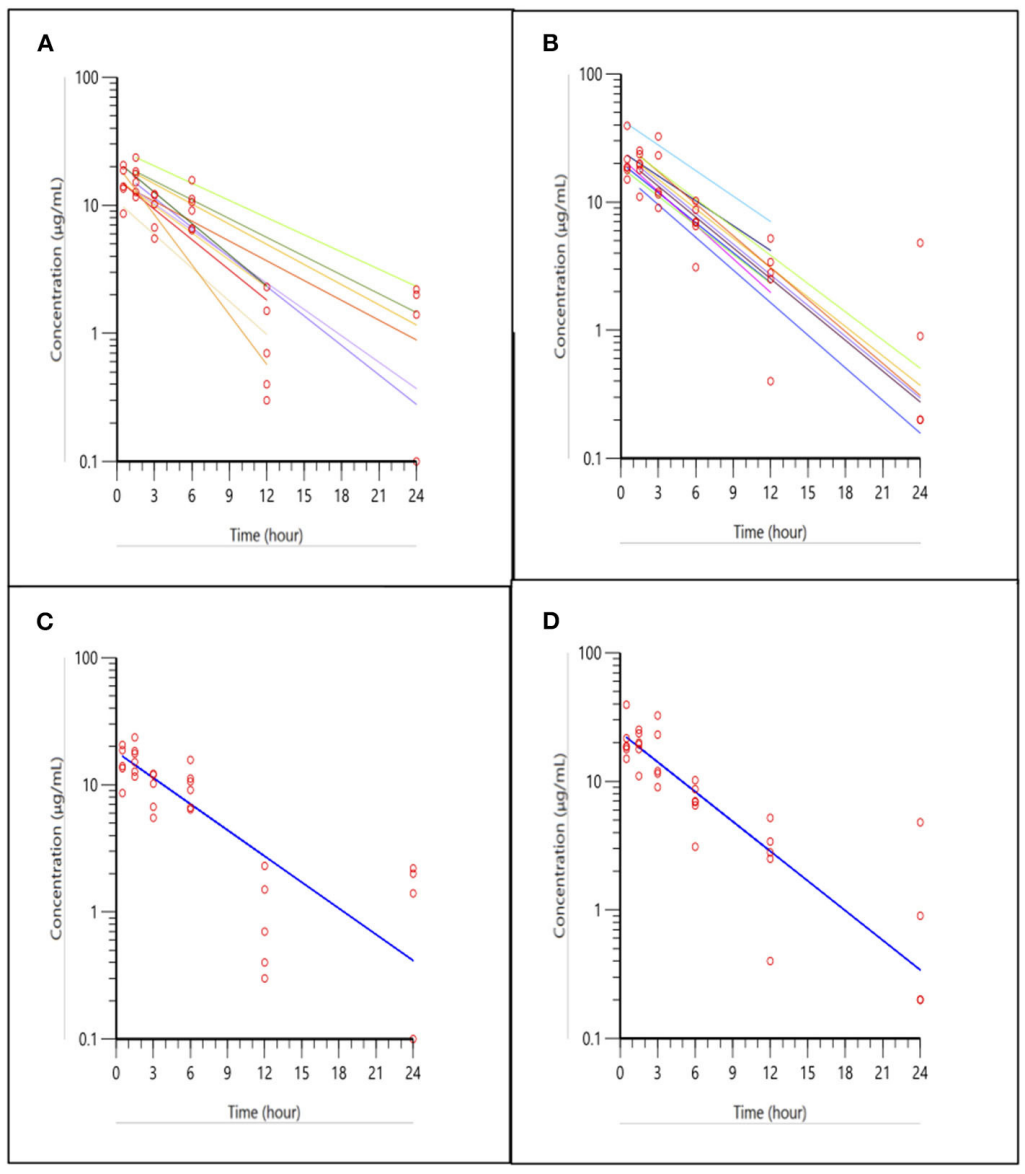
Ketoprofen concentrations in trout after IM and IV administration at 3 mg/kg. **(A,B)** shows the spaghetti plots of individual fish for IM, and IV administration, respectively, with actual points (open circles) and fitted lines (solid lines). **(A,B)** shows individual subjects fitted to the population pharmacokinetic model. Plots in **(C,D)**, (IM and IV administration, respectively) shows the fitted model for the population using a one compartment model with first order absorption, when inter-individual variability is accounted for in the model. Note the improvement in fit using the population model approach in **(C,D)**, compared to individual plots in **(A,B)**.

The pharmacokinetic curves for the 3 mg/kg ketoprofen injections in trout are presented in Figure 1, with panels A-D representing the two routes of administration (IM and IV), with spaghetti plots of individual fish (panels A and B) and the data fitted to the population model (panels C and D). The spaghetti plots show the variation among individuals and the final model for the population.

Table 1 shows the pharmacokinetic parameters for the population model of ketoprofen in trout after IM and IV injection of 3 mg/kg. Despite different routes of administration, the half-life was similar. The injection from the IM administration was almost complete as estimated from the AUC ratio of IM/IV (85% absorption) and peak concentration of 18.15 and 24.07 μg/mL from IM and IV injection, respectively. The inter-individual (between-subject) variability in concentrations from the IM injection seen in Figure 1A, is likely caused by variable IM absorption among fish, but difference in clearance cannot be excluded.

**Table 1:** Population pharmacokinetic data for ketoprofen injected in trout at a dose of 3 mg/kg, IV or IM.

**IM route 3 mg/kg**			**IV route 3 mg/kg**		
**Parameter**	**Units**	**Estimate**	**Parameter**	**Units**	**Estimate**
θV	L/kg	0.17	θV	L/kg	0.12
θKe	1/hr	0.16	θKe	1/hr	0.18
AUC	μg*hr/mL	115.24	AUC	μg*hr/mL	135.69
C_MAX_	μg/mL	18.15	C_0_	μg/mL	24.07
Cl/F	mL/hr/kg	26.03	Cl/F	mL/hr/kg	22.11
MRT	hr	6.35	MRT	hr	5.64
Half-life	hr	4.40	Half-life	hr	3.91

*The population pharmacokinetic model used non-linear mixed effects modeling (NLME) with a one-compartment, first order model. θV is the true value (theta, fixed effect) volume of distribution; θKe is the true value (theta, fixed effect) for the elimination rate constant. AUC is the area-under-the-curve for plasma concentration vs. time. C_MAX_ is the peak concentration (IM), or C_0_, the initial concentration (IV). CL, systemic clearance, or CL/F (per fraction absorbed for the IM administration. MRT, mean residence time*.

**Figure 2:**
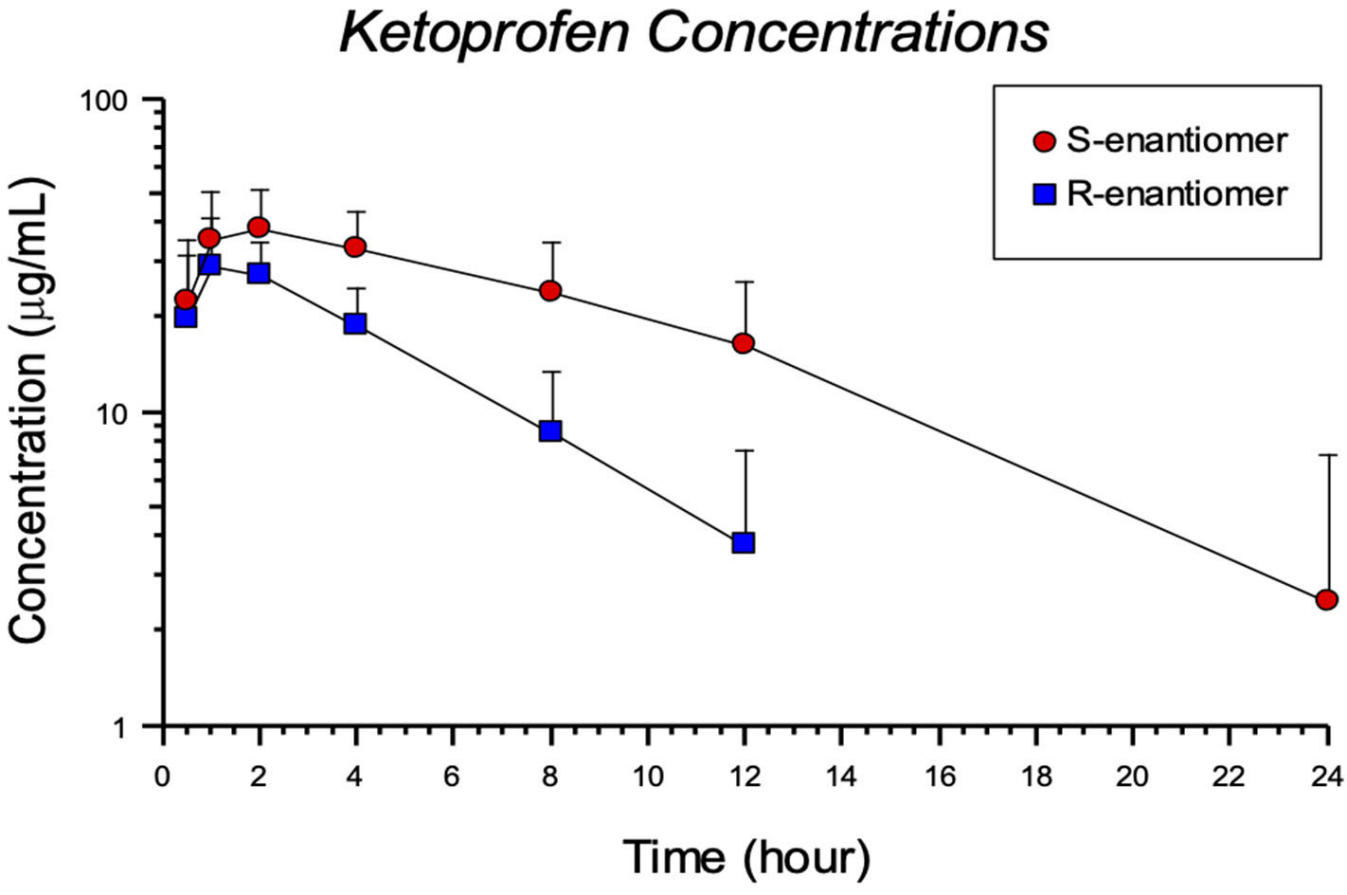
Pharmacokinetic for S and R enantiomers after an 8 mg/kg ketoprofen intramuscular (IM) injection in Nile tilapia *(Oreochromis niloticus)*.

The plasma concentrations for each enantiomer (R- and S-) after ketoprofen injection in tilapia are shown in Figure 2. Pharmacokinetic parameters for the 8 mg/kg IM ketoprofen administration to tilapia are presented in Table 2. There was a large difference between the R- and S-enantiomers, as seen by differences in the area-under-the-curve (AUC) and peak concentration (C_MAX_). The results showed a rapid absorption, but with elimination differences between the R- and S- enantiomer (Table 2). There were detectable concentrations in most fish for 24 h post injection (Figure 2). No adverse effects were observed other than some minor skin darkening around the injection site in 2 of the 12 tilapia sampled, and this resolved after the 1 hr sampling point.

**Table 2:** Summarized data after an 8 mg/kg intramuscular (IM) injection in Nile tilapia (*Oreochromis niloticus*).

**R-Enantiomer**				
**Parameter**	**Units**	**Mean**	**Std.Dev**.	**CV%**
AUC	hr*μg/mL	193.62	82.72	42.72
Clearance	mL/hr/kg	24.41	11.96	48.99
Cmax	μg/mL	30.23	10.52	34.79
K01	1/hr	1.80	1.79	99.14
Absorption half-life	hr	0.64	0.44	68.14
K10	1/hr	0.28	0.14	50.30
Elimination half-life	hr	3.10	1.47	47.32
Tmax	hr	1.60	0.55	34.64
VD/F	mL/kg	98.11	41.01	41.80
**S-Enantiomer**				
**Parameter**	**Units**	**Mean**	**Std.Dev**.	**CV%**
AUC	hr*μg/mL	510.37	284.54	55.75
Clearance	mL/hr/kg	10.29	6.44	62.57
Cmax	μg/mL	40.83	13.36	32.72
K01	1/hr	4.80	8.89	185.08
Absorption half-life	hr	0.82	0.74	90.41
K10	1/hr	0.15	0.10	65.97
Elimination half-life	hr	6.81	4.37	64.09
Tmax	hr	2.13	1.14	53.56
VD/F	mL/kg	80.16	36.31	45.30

## Discussion

This is the first reported analysis of the pharmacokinetics of the NSAID ketoprofen in teleost fish. Ketoprofen has been assessed as an analgesic in some fish (12, 14, 25). A minimum gill concentration model (akin to minimum alveolar concentration in traditional mammalian anesthesia studies) was established in rainbow trout (*Oncorhynchus mykiss*), with a noxious stimulus applied under varying dosages of butorphanol and ketoprofen against varying concentrations of MS-222 (14). The same model was used in the chain dogfish (*Scyliorhinus retifer*) (with a change of terminology from MGC to minimum anesthetic concentration (MAC) but this approach did not show a change in MAC to any ketoprofen or MS-222 dosage used (25). A similar MAC response model was also assessed in goldfish using a ketoprofen dosage of 0.5–2 mg/kg IM and in this case, the drug was found to significantly decrease the MAC (16). Another study showed that ketoprofen at 2 mg/kg reduced muscle damage post-surgery in koi (12). No adverse effects were reported in any of these studies. Interestingly, when comparing the risks of ketoprofen and diclofenac in rainbow trout, ketoprofen was determined to be the safer of the two drugs, as diclofenac alters organ histology and gene expression in fish at 1 μg/L (30).

Ketoprofen is formulated as a racemic mixture, containing equal (50:50) amounts of each R- and S- enantiomers (23). Understanding the pharmacokinetics of each enantiomer can provide information about whether potential therapeutic efficacy and/or toxic effects can be attributed to one enantiomer over the other (22, 23, 31). The S-enantiomer of ketoprofen is associated with anti-prostaglandin activity and toxicity, while the R-enantiomer is associated with analgesia and has no interaction with the COX binding site (32). Additionally, the S-enantiomer is absorbed rapidly from the gastrointestinal tract and is characterized by a quick onset of biological action (33). In the present study, exposure was much greater for the S-enantiomer in teleosts compared to the R-, which is similar to findings in studies with horses, dogs, and cats (31, 32, 34–37). R- and S-enantiomers were analyzed separately in the present study for tilapia but they were not differentiated for trout. It is unknown which form is the most active in teleosts but it has been found the S-enantiomer is the most active for therapeutic effects in mammals (22–24, 39). One study assessed both enantiomers in freshwater fish (*Alburnus alburnus, Lepomis gibbosus, Micropterus salmoides, Oncorhynchus mykiss*, and *Cyprinus carpio*) and found no statistically significant difference in their concentration present in fish tissue (38). In this study, the volume of distribution was similar between both isomers.

For trout, the half-life of ketoprofen fell in-between the values reported for both the R- and S- in the tilapia, therefore, it may represent a contribution from both enantiomers. The clearance was similar between trout and tilapia, but the volume of distribution was lower in the tilapia. A relevant study in freshwater fish hepatic cell-lines found that the racemic mixture and the S-enantiomer may have acted through different mechanisms within cells and resulted in different responses (40). In the present study, the differences observed between the R- and S-enantiomers represents either more rapid clearance of the R-enantiomer, or chiral inversion from R- to the S-enantiomer, which occurs in mammals (22, 23, 41, 42).

There were some limitations to this study that are important to address. Necessary, repeat handling of animals is atypical when compared to other fish pharmacological studies. However, the origin and disposition of the animals in the present study were unique and intended for animals in aquaria where individuals are managed vs. a school. Additionally, repeat sampling is typical of similar studies in different taxa. MS222 was used as an anesthetic in trout, but not tilapia. With a fixed number of animals available and their welfare in mind, handling was designed to acquire samples with the least amount of anticipated stress for each species and their perceived levels of tolerance to restraint stress. The use of anesthetics has been shown to reduce stress in teleosts (43–45), although responses are varied and contingent on species and circumstance (46, 47). While MS222 can induce cortisol release and alter some other biochemical values in trout (47–50), it has been shown that it reduces the stress response overall (45). Others may argue that anesthesia is stressful to the animal and could result in subsequent physiological effects (51). Thus, when handling time is minimal and procedures are minimally or non-invasive, the addition of anesthesia and increased total time of handling may not be needed (52). Tilapia have also been shown to respond more slowly to MS222 (53) and require high levels of sedation (54). Author personal experience (Burns, Mylniczenko) noted that the time and relatively high dose of MS222 required for this species for adequate anesthetic induction would be greater than the handling time required for sample collection and in that regard, was considered less beneficial to reduce stress. Additionally, handling techniques with skilled personnel were ensured to be rapid and without trauma. Thus, the use of anesthesia for handling in minimally invasive procedures in teleosts is a continued debate and needs to be assessed on a case by case basis considering staff experience, facility capacity, and species tolerance (46). Ideally, pharmacokinetic studies in fish should explore both manual restraint vs. anesthesia, as well as animal stress levels to fully understand the impact on drug kinetics. Finally, between subject variability was observed in the present study as has been seen in other studies (47, 55). Fluid shifts from the secondary vascular system and stress (56) could account for this variability but were out of the scope to explore for the current project and warrant future investigation.

The results from the present study provide pharmacokinetic data for ketoprofen at a dosage of 3 and 8 mg/kg in two teleost species. There were only minor adverse effects observed and no major effects at either dosage in any of the fish. Humane veterinary practice dictates the incorporation of appropriate analgesics and anti-inflammatories into medical treatment strategies for fish (14, 17, 18). Though it cannot be inferred that these doses provided analgesia, they did reach what would be therapeutic levels in mammals. Additionally, at once-daily dosing the drug remained in the body for at least 24 h post injection in most fish, which makes it reasonable for clinical use in fish medicine.

## Data Availability Statement

All datasets generated for this study are included in the article/supplementary material.

## Ethics Statement

The animal study was reviewed and approved by Mote Marine Laboratory and Aquarium Institutional Animal Care and Use Committee (IACUC).

## Author Contributions

WG: project design, development, sample collection (tilapia), and manuscript writing. NM: project design, development, sample collection (trout), and manuscript writing and mentoring. TS: project design, development, sample collection (trout), and manuscript writing. CB: project design, sample collection (tilapia), and manuscript writing. GL: project development (tilapia), mentoring, and manuscript writing. LB: sample collection and processing (tilapia). MP: sample analysis, pharmacokinetics, and manuscript writing. All authors contributed to the article and approved the submitted version.

## Conflict of Interest

The authors declare that the research was conducted in the absence of any commercial or financial relationships that could be construed as a potential conflict of interest.
